# Categorization of post-cardiac arrest patients according to the pattern of amplitude-integrated electroencephalography after return of spontaneous circulation

**DOI:** 10.1186/s13054-018-2138-2

**Published:** 2018-09-20

**Authors:** Kazuhiro Sugiyama, Kazuki Miyazaki, Takuto Ishida, Takahiro Tanabe, Yuichi Hamabe

**Affiliations:** Tertiary Emergency Medical Center, Tokyo Metropolitan Bokutoh Hospital, 23-15 Kohtohbashi, 4-Chome, Sumida-ku, Tokyo, 130-8575 Japan

**Keywords:** Hypoxic encephalopathy, Amplitude-integrated electroencephalography, Prognostication, Post-cardiac arrest care

## Abstract

**Background:**

Continuous electroencephalography (cEEG), interpreted by an experienced neurologist, has been reported to be useful in predicting neurological outcome in adult patients post cardiac arrest. Amplitude-integrated electroencephalography (aEEG) is a type of quantitative EEG and is easily interpreted by a non-neurologist. A few studies have shown the effectiveness of aEEG in prognostication among adult patients post cardiac arrest. In this study, we hypothesized that the pattern of aEEG after return of spontaneous circulation (ROSC) could successfully categorize patients post cardiac arrest according to their expected neurological outcome.

**Methods:**

We assessed the comatose survivors of out-of-hospital cardiac arrest who received targeted temperature management with midazolam-based sedation and were monitored with aEEG at our tertiary emergency care center from January 2013 to June 2017. We categorized the patients into categories 1 (C1) to 4 (C4). C1 included patients who regained continuous normal voltage (CNV) within 12 h post ROSC, C2 included those who recovered CNV 12–36 h post ROSC, C3 included those who did not recover CNV before 36 h post ROSC, and C4 included those who had burst suppression at any time post ROSC. We evaluated the outcomes of neurological function for each category at hospital discharge. A good outcome was defined as a cerebral performance category of 1 or 2.

**Results:**

A total of 61 patients were assessed (median age, 60 years), among whom 42 (70%) had an initial shockable rhythm, and 52 (85%) had cardiac etiology. Of all 61 patients, 40 (66%) survived to hospital discharge and 27 (44%) had a good neurological outcome. Of 20 patients in C1, 19 (95%) had a good outcome, while the percentage dropped to 57% among C2 patients. No patients in C3 or C4 had a good outcome. Three patients could not be classified into any category.

**Conclusions:**

The pattern of aEEG during the early post-cardiac-arrest period can successfully categorize patients according to their neurological prognoses and could be used as a potential guide to customize post-cardiac-arrest care for each patient.

**Electronic supplementary material:**

The online version of this article (10.1186/s13054-018-2138-2) contains supplementary material, which is available to authorized users.

## Background

Predicting the neurological outcome of patients post cardiac arrest during target temperature management (TTM) remains a challenge. Current guidelines recommend continuous electroencephalography (cEEG) in these patients for seizure management [1, 2]. Moreover, the pattern of conventional EEG or cEEG is also thought to be a useful prognostic tool and a large number of studies have reported the effectiveness of these tests in predicting both good and poor prognoses [3–6]. However, cEEG is difficult for non-neurologists to interpret. Intensivists or emergency physicians, who treat survivors of cardiac arrest, require prognostic markers that they could assess at the bedside to customize post-cardiac-arrest care for each patient. Amplitude-integrated electroencephalography (aEEG) is a type of quantitative EEG and is usually derived from single-channel or two-channel cEEG recordings. The maximum and minimum amplitudes within a short period are displayed as bandwidths along with a compressed time scale. aEEG is easily interpreted by intensivists and emergency physicians at the bedside. Recently, the pattern of aEEG after return of spontaneous circulation (ROSC) has been reported to be useful for predicting neurological outcomes in adult patients post cardiac arrest [7–9]. The early recovery of a normal pattern of aEEG after ROSC is reported to be a predictor of good neurological outcome. On the other hand, the absence or late recovery of a normal trace, or the presence of burst suppression, suggests a poor neurological outcome. In the present study, we hypothesized that the pattern of aEEG after ROSC could be used to categorize patients post cardiac arrest according to their expected neurological outcome, making it a useful tool for personalizing the care of these patients.

## Methods

### Patients

This retrospective study included comatose survivors of out-of-hospital cardiac arrest who were treated with TTM and monitored with aEEG after ROSC at the tertiary emergency care center of Tokyo Metropolitan Bokutoh Hospital between January 2013 and June 2017. Some of the patients in this study were also included in our previous study [7]. Patients younger than 18 years and patients in whom aEEG monitoring was not started within 12 h after ROSC were excluded. The baseline demographic and clinical characteristics of the patients were collected from their medical records, and the timing of pre-hospital events were recorded according to the reports of emergency medical service personnel. The institutional review board of Tokyo Metropolitan Bokutoh Hospital approved the study (institutional approval reference number 29–54), which complied with the tenets of the Declaration of Helsinki. The requirement of informed consent was waived, owing to the retrospective design of the study.

### TTM protocol and post-resuscitation care

All patients were resuscitated according to the current recommendations. The patients received appropriate amounts of fluid or vasopressors to maintain the mean blood pressure above 65 mmHg, and were ventilated to maintain normocarbia and prevent hypoxia and hyperoxia (partial arterial pressure of oxygen (PaO_2_) > 300 mmHg). Patients who were suspected to have cardiac etiology underwent coronary angiography and percutaneous coronary intervention if indicated. Patients who remained comatose after ROSC were treated with TTM at 34 °C for 24 h. The exclusion criteria for TTM included hemodynamic instability refractory to the use of vasopressor agents and mechanical support, refractory ventricular arrhythmia, active bleeding, and terminal illness prior to the cardiac arrest. TTM was started immediately after admission to the emergency department and was applied by using a surface cooling device (Arctic Sun System; Medivance, Louisville, CO, USA). Patients who experienced witnessed cardiac arrest, who had initial shockable rhythm and were aged ≤ 65 years underwent extracorporeal cardiopulmonary resuscitation (ECPR) if the cardiac arrest was sustained and refractory to conventional advanced life support procedures after hospital arrival. A heat exchanger was used in the circuit to control body temperature in these patients. Bladder temperature was used for temperature management. Midazolam (0.05–0.1 mg/kg body weight/h) and fentanyl (1 μg/kg body weight/h) were administered for sedation and analgesia, and vecuronium (0.05–0.1 mg/kg body weight/h) was administered to prevent shivering. After maintaining the temperature at 34 °C for 24 h, the patients were rewarmed at the rate of 0.15 °C/h until the body temperature reached 36 °C, after which midazolam and vecuronium were discontinued.

Neurological outcomes were predicted based on the clinical examinations performed at least 72 h post ROSC and on brain computed tomography (CT) findings. Patients’ neurological outcomes were predicted as poor if they remained unconscious at least 72 h post ROSC, had a motor response component of the Glasgow Coma Scale (GCS) score ≤ 2, were without pupillary reflex, and had apparent loss of the border of white and gray matter or diffuse brain swelling recognized on initial or follow up (on days 4–5) brain CT. Even in these patients, we did not withdraw treatment, and ongoing life-sustaining measures were continued. However, additional aggressive treatment modalities such as hemodialysis and additional mechanical circulatory support devices were withheld.

### aEEG data

**Fig. 1:**
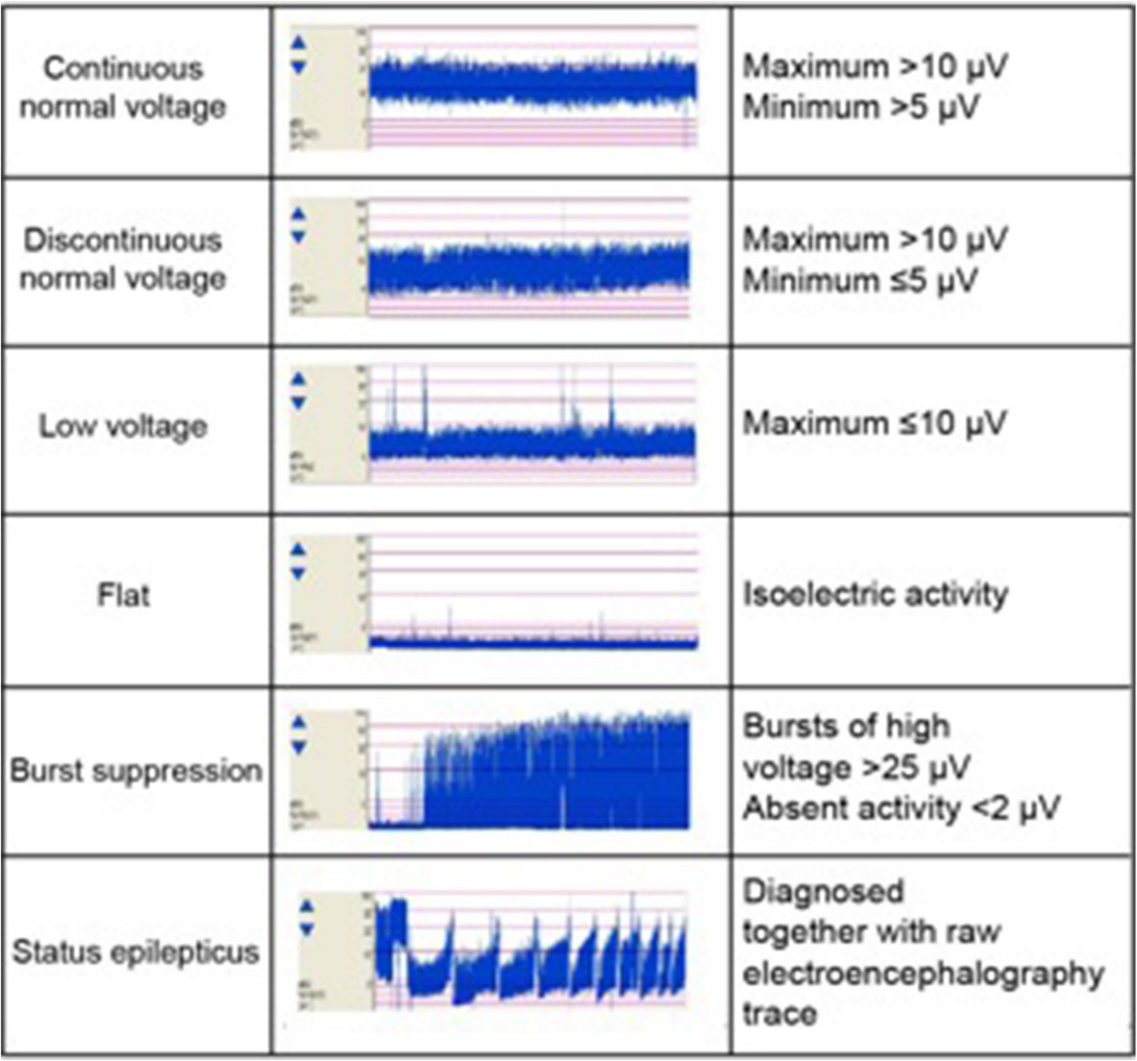
Classification of the patterns of amplitude-integrated electroencephalography traces used in the study

All patients were monitored with aEEG after admission to the intensive care unit. aEEG monitoring was performed using the bipolar channel Fp1–Fp2 (Additional file 1: Figure S1). We applied the classification system of aEEG patterns used by Oh et al. (Fig. 1) [10]. In this classification, a continuous normal voltage (CNV) trace (maximum amplitude > 10 μv and minimum amplitude > 5 μv) was considered the best trace for patients post cardiac arrest. The CNV recovery time was defined as the duration from ROSC to the first time when a CNV trace was recorded. When a CNV was transient, the time of the first voltage was recorded provided it was distinguishable from noise with reference to the raw trace of EEG.

Burst suppression was diagnosed based on the trace of aEEG, which was confirmed with the raw trace to meet the criteria of the American Clinical Neurophysiology Society (ACNS) that “more than 50% of the EEG record should consist of periods of EEG voltage <10 μV, with alternating bursts” [11].

Electrographic status epilepticus (ESE) was diagnosed using the criteria reported by Rundgren et al. [9]. In diagnosing ESE, the raw trace of cEEG was referenced together with the trace of aEEG.

### Outcomes

The cerebral performance category (CPC) scale was used to assess the outcomes of neurological function at hospital discharge [12]. The CPC scores were determined by a review of the report from the rehabilitation department. The outcome of neurological function was considered good if the CPC score was 1 or 2, and poor if the CPC score was 3–5.

### Categorization of patients post cardiac arrest

We categorized the patients post cardiac arrest into four groups (C1–C4) according to the CNV recovery time and the presence of burst suppression. C1, C2, C3, and C4 included patients who recovered CNV within 12 h after ROSC, patients who recovered CNV between 12 and 36 h after ROSC, patients who did not exhibit CNV within 36 h after ROSC, and patients who exhibited burst suppression at any time after ROSC, respectively.

### Statistical analysis

Continuous variables are reported as medians with interquartile ranges (IQRs), and dichotomous variables are reported as numbers with percentages. The chi-square test for categorical variables and the Kruskal-Wallis test for continuous variables were used to compare the differences in patient characteristics between the categories. All statistical analyses were performed using EZR (Saitama Medical Center, Jichi Medical University, Saitama, Japan) [13].

## Results

**Fig. 2:**
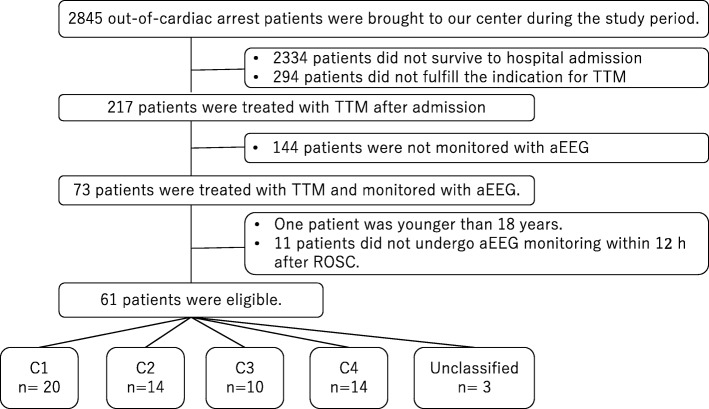
Flow chart of patient selection in the study. TTM, target temperature management; aEEG, amplitude-integrated electroencephalography; ROSC, return of spontaneous circulation; C1, C2, C3, C4, categories 1, 2, 3, and 4.

During the study period, a total of 2845 patients with out-of-hospital cardiac arrest were transferred to our center, and 217 patients were treated with TTM after successful resuscitation. Of these patients, 144 were not monitored with aEEG because the monitoring device was occupied by other patients at admission. Therefore, 73 patients were analyzed, 12 of whom were excluded, leaving 61 eligible patients. Among these patients, 20 patients were classified into C1, 14 into C2, 10 into C3, and 14 into C4. Three patients were not classified into any category (Fig. 2).

The characteristics of all patients in each category are shown in Table 1. Of the total 61 patients, 51 (84%) were male. The median age of the patients was 60 (IQR, 46–68) years, and collapse was witnessed in 57 patients (93%). A total of 52 (85%) patients had cardiac etiology, among whom 14 (23%) patients suffered from acute coronary syndrome. A total of 19 patients (31%) underwent ECPR, and 54 patients (89%) underwent coronary angiography. The median time from collapse to ROSC was 23 (IQR, 18–39) min. The median time from ROSC to the start of aEEG monitoring was 5.2 (IQR, 4–7.1) h, and the median duration of aEEG monitoring was 53 (IQR, 46–65) h. The proportions of patients with initial shockable rhythm, the GCS scores and the positive pupillary reflex at the start of TTM, the time from collapse to ROSC, and the proportions of patients undergoing ECPR, were significantly different among the categories.

**Table 1:** Patient characteristics in each category (C)

	All patients	C1	C2	C3	C4	
	*n* = 61	*n* = 20	*n* = 14	*n* = 10	*n* = 14	*p* value
Age (years)^a^	60 (46–68)	50 (44–63)	66 (52–72)	66 (45–69)	63 (58–71)	0.11
Male, *n* (%)	51 (84%)	15 (75%)	10 (71%)	10 (100%)	14 (100%)	0.09
Witnessed collapse, *n* (%)	57 (93%)	19 (95%)	12 (86%)	10 (100%)	13 (93%)	0.67
Initial shockable rhythm, *n* (%)	42 (70%)	16 (84%)	13 (93%)	6 (60%)	7 (50%)	0.02
Time from collapse to ROSC (min)^a^	23 (18–39)	19 (15–22)	22 (17–29)	46 (38–49)	39 (23–44)	< 0.01
Cardiac etiology, *n* (%)	52 (85%)	18 (90%)	13 (93%)	9 (90%)	11 (79%)	0.66
Acute coronary syndrome, *n* (%)	14 (23%)	2 (10%)	3 (21%)	4 (40%)	4 (29%)	0.28
GCS at admission	3 (3–3)	4 (3–6)	3 (3–4)	3 (3–3)	3 (3–3)	< 0.01
Pupillary reflex at admission, *n* (%)	29 (48%)	15 (75%)	8 (57%)	2 (20%)	2 (14%)	< 0.01
Coronary angiography, *n* (%)	54 (89%)	18 (90%)	14 (100%)	9 (90%)	11 (79%)	0.33
ECPR, *n* (%)	19 (31%)	4 (20%)	1 (7.1%)	7 (70%)	5 (36%)	0.01
Time from collapse to initiation of ECMO flow in ECPR patients (min)*	39 (15–56)	28 (15–53)	37	45 (33–55)	48 (38–56)	0.13

*ROSC* return of spontaneous circulation, *ECPR* extracorporeal cardiopulmonary resuscitation, *GCS* Glasgow coma scale

^a^Median (interquartile range)

**Fig. 3:**
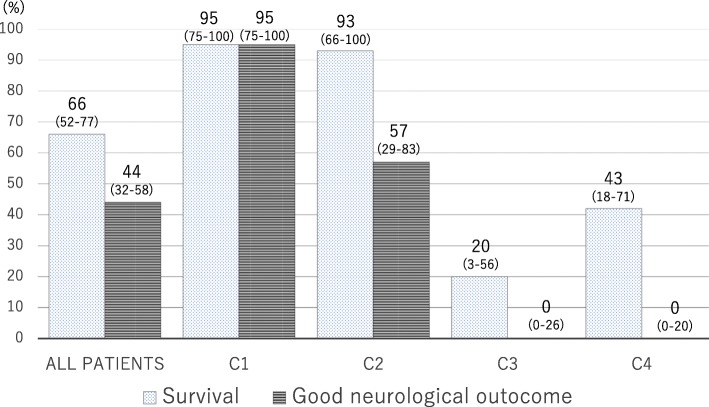
Survival and outcomes of neurological function for each category (C). Of the 20 patients in C1, 19 (95%) survived with good neurological outcomes. Of the 14 patients in C2, 13 (93%) survived and 8 (57%) had good neurological outcomes. The survival rates in C3 and C4 were low, and no patients had good neurological outcomes. The 95% confidence intervals are shown under each percentage

Of all 61 patients, 40 (66%) survived to hospital discharge and 27 (44%) had good neurological outcomes. Of the 20 patients in C1, 19 (95%) survived, with good neurological outcomes. Of the 14 patients in C2, 13 (93%) survived and 8 (57%) had good neurological outcomes. The survival rates in C3 and C4 were low, and no patients had good neurological outcomes (Fig. 3).

Clinical examination findings (motor response of the GCS and pupillary reflex) at 72 h after ROSC and the findings of diffuse anoxic injury on brain CT in each category are shown in Table 2. One patient in C1 died from circulatory failure after regaining consciousness and was able to obey simple commands. One patient in C2 died on day 12 from recurrent cardiopulmonary collapse without definite neurological prognostication. Of the eight patients who died in C3, six died after their neurological prognoses were predicted as poor based on the clinical examination 72 h post ROSC and on brain CT findings. Two patients died within 72 h without definite neurological prognostication; however, one of them exhibited anoxic injury on initial brain CT. Eight patients died in C4 after their neurological prognoses were predicted as poor.

**Table 2:** Neuro-prognostication tests in each category (C)

	C1	C2	C3	C4
	*n* = 20	*n* = 14	*n* = 10	*n* = 14
Clinical examination at 72 h post ROSC				
Patients who survived for > 72 h	20	14	8	14
Motor response of GCS ≤ 2, *n* (%)	1/20 (5%)	4/14 (29%)	8/8 (100%)	14/14 (100%)
Negative pupillary reflex, *n* (%)	0	2/14 (14%)	6/8 (75%)	8/14 (57%)
Diffuse anoxic injury on brain CT^a^, *n* (%)	0	2/14 (14%)	7/10 (70%)	11/14 (79%)

*ROSC* return of spontaneous circulation, *GCS* Glasgow coma scale, *CT* computed tomography

^a^Apparent loss of the border of white and gray matter or diffuse brain swelling on initial or follow up brain CT

Of the 10 patients in C3, 2 patients recovered CNV at 41 h and 74 h after ROSC, respectively. Of 14 patients in C4, 13 exhibited burst suppression within 24 h after ROSC.

Overall, seven patients (11%) exhibited ESE during the aEEG monitoring. No patient in C1 had ESE, while three patients (21%) in C2 and three patients in C4 (21%) had ESE. Among these patients with ESE, only one patient in C2 had a good neurological outcome.

## Discussion

In the present study, we categorized the patients post cardiac arrest into 4 categories based on the pattern of aEEG after ROSC, using the CNV recovery time and the presence of burst suppression. These patients were successfully categorized into those with excellent neurological prognosis (C1), intermediate neurological prognosis (C2), and very poor prognosis (C3, C4). Some variables of quantitative EEGs other than aEEG, such as burst suppression ratio, spectral variability and response entropy, have been reported to be associated with prognosis in patients post cardiac arrest [14–16]. However, to the best of our knowledge, classification of adult patients post cardiac arrest based on the pattern of their aEEG and the severity of hypoxic encephalopathy has not been suggested before. Furthermore, this categorization can be made at the bedside by the intensivist or emergency physician without input from an experienced neurophysiologist.

aEEG has already been widely used in the field of neonatal intensive care, and been reported to be useful to predict the neurological outcome of hypoxic-ischemic encephalopathy in neonates [17]. The greatest advantage of aEEG is its ease of interpretation, and the inter-observer difference has been reported to be acceptable in evaluating background activity and detecting status epilepticus [18–20]. In this study, we used the frontal bipolar lead. Using this method, aEEG monitoring is very easy to set up and can be started rapidly. It is suitable in the situation of post-cardiac-arrest care, where numerous procedures are needed. Though focal seizures might be missed during monitoring with a frontal hairline lead, the global function of the brain can be evaluated [8, 10].

The duration from ROSC to the recovery of a CNV trace has been reported to be associated with the severity of hypoxic encephalopathy in adult patients post cardiac arrest treated with TTM. Oh et al. reported that the cutoff for the CNV recovery time to predict a good neurological outcome was 24 h [8]. In our previous study, this cutoff was similarly found to be 23 h [7]. However, though a CNV recovery time around 24 h had good sensitivity and specificity to predict a good outcome, some patients within this timeframe still had poor neurological outcomes in both studies. Hence, in the present study, we aimed to identify patients post cardiac arrest who had intact cerebral function or very mild hypoxic encephalopathy, to set the cutoff value of C1 to within 12 h after ROSC. Further, in a previous study using cEEG, Tjepkema-Cloostermans et al. reported that a normal or diffusely slow electroencephalogram at 12 h after cardiac arrest could predict a good neurological outcome, with specificity of 96% [6]. Although several predictors have been reported to be useful for predicting poor neurological outcome in patients post cardiac arrest [1, 2], predictors that can foretell a good neurological outcome are scarce. Accordingly, this is the greatest advantage of using aEEG monitoring in post-cardiac-arrest care; intensivists or emergency physicians can identify the patients with potential good neurological outcome at the bedside. On the other hand, late recovery of the CNV trace predicts poor neurological outcome. Oh et al. reported this cutoff as 36 h [8].

Burst suppression has been reported as a predictor of poor neurological outcome in many previous studies [3–5]. However, some studies have reported on neurological recovery in patients with burst suppression during TTM [21, 22]. Amorim et al. reported that 8 of 22 patients with burst suppression had good neurological outcomes [23]. Also, the current European Resuscitation Council guidelines mentioned that burst suppression may be compatible with neurological recovery within 72 h after ROSC [2]. In this study, all patients in C4 exhibited burst suppression within 72 h and had poor neurological outcomes. In other studies that evaluated the efficacy of aEEG in prognostication, burst suppression also predicted a poor neurological outcome with very high specificity [8, 9].

The definition and kind of sedative used make it difficult to evaluate the efficacy of burst suppression in prognostication. Although the definition of burst suppression in cEEG has been suggested by the ACNS recently, most previous studies did not comply with this definition [2, 11]. In our study, burst suppression was diagnosed by initially evaluating the trace of aEEG, and was then confirmed with the raw trace to meet the ACNS criteria. This approach could lead to the stricter identification of burst suppression compared to evaluating cEEG alone. Unlike our study, propofol was used for sedation in many patients in the study conducted by Amorim et al., and it is known that propofol might induce burst suppression. As such, the relatively high number of patients with burst suppression among those with good neurological outcomes seen in their study might simply reflect the effect of propofol. Although midazolam has also been reported to induce burst suppression in term infants without brain injury [24], the prognostic value of burst suppression might differ among the studies that used propofol and midazolam as sedatives during TTM. Our study suggests that patients who were sedated with midazolam and exhibited burst suppression in the early phase after ROSC could have severe hypoxic encephalopathy. However, further studies are needed to determine the efficacy of burst suppression as a predictor of poor outcome.

The categorization system used in this study could help us customize post-cardiac-arrest care for each patient. In this study, patients in C1 were found to have an excellent neurological prognosis. These patients had intact cerebral function or subtle hypoxic encephalopathy. Every possible invasive treatment should be performed for these patients when warranted, even if they remain comatose when the highly invasive or high-cost treatment would be performed. Moreover, the post-cardiac-arrest care among these patients could possibly be simplified. TTM is not free from complications such as infection, circulatory collapse, arrhythmia, and bleeding; in these patients, the duration of TTM could potentially be shortened to less than 24 h, and we could awaken the patients earlier and concentrate on treating the cause of the cardiac arrest. We believe that it is worth further evaluating this approach in a future study. Patients in C2 were shown to have a borderline neurological prognosis and are thus considered to be optimal targets for intensive post-cardiac-arrest neurological care. Status epilepticus was observed among these patients, and one such patient had a good neurological outcome in this study. Some patients with status epilepticus post cardiac arrest have been reported to have good neurological outcomes if status epilepticus occur from a continuous background [9]. Accordingly, aggressive monitoring and management of status epilepticus should be performed. Conversely, patients in C3 and C4 were found to have very poor prognoses. Besides the aEEG findings, they had other characteristics associated with poor prognosis. Patients in C3 and C4 underwent prolonged cardiopulmonary resuscitation (CPR) and fewer of them had initial shockable rhythm, and patients in C3 had underwent more ECPR, which is usually associated with worse outcomes. The damage to the brain is considered very severe in these patients. However, further studies are required before these criteria for aEEG can be used for treatment withdrawal, and for now, neuro-prognostication should be made based on the multimodal approach after allowing sufficient time.

Recently, some treatment approaches against hypoxic encephalopathy, which were previously considered promising, have failed to show effectiveness. For example, TTM at 33 °C was not shown to be superior to TTM at 36 °C [25], and a TTM duration of 48 h was not superior to 24 h in terms of the neurological outcome of the patients [26]. Proper patient selection will affect the effectiveness of a certain treatment. In other words, patients with very mild and extremely severe injuries should be excluded. Such a classification of severity has not been established in patients post cardiac arrest, and both of the aforementioned studies included patients with different severity of hypoxic encephalopathy. The categorization used in the present study can help identify patients with mild, moderate, and severe hypoxic encephalopathy, and thus could be used as a tool for the stratification of patients in clinical trials.

The main disadvantage of this proposed categorization is the time it takes to prepare it. Ideally, earlier timing of prognostication of hypoxic encephalopathy after ROSC is desired. In fact, it takes at least a few hours to categorize patients in C1, and even longer to categorize patients in C2 and C3. However, we believe that it is still worth using this categorization, because early identification of patients with good neurological outcome is very useful even after several hours post ROSC, and this system would also allow the customization of post-cardiac-arrest care according to the pattern of aEEG as time goes by after ROSC. Of note, in patients in C3 and C4, sufficient time must pass to consider withdrawing or withholding treatment.

Three patients could not be classified into any category in this study; they all exhibited flat patterns, but the monitoring periods were shorter than 36 h. Had they had sufficiently longer monitoring periods, they would most likely have been categorized.

This study had some important limitations. First, this was a retrospective single-center study with a small sample size, and not all patients treated with TTM were monitored with aEEG during the study period because of the shortage of monitoring devices. Although all consecutive patients monitored with aEEG were included in this study, this could lead to selection bias. Furthermore, this study included many patients treated with ECPR, leading to the relatively low rate of good neurological outcome compared with other studies. Second, outcomes of neurological function were evaluated at hospital discharge; thus, the long-term prognosis was not evaluated. The median duration of stay hospital in hospital by patients with a CPC score of 3 or 4 was 55 (IQR, 43–68) days. This is relatively long, but not long enough. Tong et al. reported that a significant proportion of patients with poor neurological outcomes at hospital discharge experienced improvement in their neurological statuses over several months post discharge [27]. In this study, two patients in C3 had CPC scores of 3 at hospital discharge. These patients might have the potential to improve their neurological status during long-term follow up. Third, all eligible patients were sedated with midazolam. Therefore, the results might be different in patients treated with other sedatives, as CNV recovery time might be influenced by the kind of sedative used, and the possibility of propofol-induced burst suppression should not be ignored. Fourth, the physicians were not blinded to the results of the pattern of aEEG. However, no patient required withdrawal or withholding of therapy based on the pattern of aEEG in this study. Fifth, the sensitivity of status epilepticus might not be sufficient compared with that in other studies, because we only used the frontal lead in this study.

## Conclusions

The pattern of aEEG after ROSC can successfully categorize patients post cardiac arrest according to their neurological prognoses and could be used as a guide to customize post-cardiac-arrest care for each patient.

## Additional file

Additional file 1: **Figure S1.** Placement of electrodes for amplitude-integrated electroencephalography (aEEG) monitoring. Cup or hydrogel electrodes were attached at positions Fp1 and Fp2, and aEEG monitoring was performed for the bipolar channel Fp1–Fp2. (JPG 14 kb)

## Abbreviations

aEEG: Amplitude-integrated electroencephalography
cEEG: Continuous electroencephalography
CNV: Continuous normal voltage
CPC: Cerebral performance category
CT: Computed tomography
ECPR: Extracorporeal cardiopulmonary resuscitation
ESE: Electrographic status epilepticus
GCS: Glasgow Coma Scale
IQR: Interquartile range
ROSC: Return of spontaneous circulation
TTM: Target temperature management
