# Supplementary figures and images for: Categorization of post-cardiac arrest patients according to the pattern of amplitude-integrated electroencephalography after return of spontaneous circulation
Source: Crit Care. 2018 Sep 20;22:226. doi: 10.1186/s13054-018-2138-2 (PMC6148786; doi:10.1186/s13054-018-2138-2)

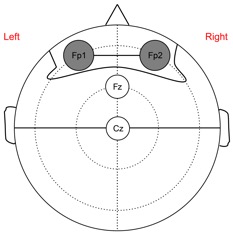

Supplement: Supplementary file 1 — Figure S1. Placement of electrodes for amplitude-integrated electroencephalography (aEEG) monitoring. Cup or hydrogel electrodes were attached at positions Fp1 and Fp2, and aEEG monitoring was performed for the bipolar channel Fp1–Fp2. (JPG 14 kb) [file 13054_2018_2138_MOESM1_ESM.jpg]
